# Infant feeding practices in three Latin American countries in three decades: what demographic, health, and economic factors are relevant?

**DOI:** 10.3389/fnut.2023.1239503

**Published:** 2023-10-04

**Authors:** Camila Abadia Rodrigues Meira, Gabriela Buccini, Catarina Machado Azeredo, Wolney Lisbôa Conde, Ana Elisa Madalena Rinaldi

**Affiliations:** ^1^School of Medicine, Federal University of Uberlândia, Uberlândia, Brazil; ^2^School of Public Health, University of Nevada, Las Vegas, NV, United States; ^3^School of Public Health, University of São Paulo, São Paulo, Brazil

**Keywords:** child nutrition, breastfeeding, demographic factors, economic factors, Latin America, health

## Abstract

**Introduction:**

Studies in Latin America have focused either on analyzing factors associated with exclusive breastfeeding (EBF) or infant formula (IF).

**Purpose:**

Analyze the association between economic, sociodemographic, and health factors with EBF, mixed milk feeding (MixMF), and exclusive use of IF in three Latin American and Caribbean countries in the 1990s, 2000s, and 2010s.

**Methods:**

Cross-sectional time-series study using data from Demographic and Health Surveys between the 1990s and 2010s in Colombia (1995–2010), Haiti (1994–2017), and Peru (1996–2012) accounting for a sample of 12,775 infants under 6 months. Hierarchical logistic multilevel regression models were used to estimate the adjusted association between infant feeding outcomes (EBF, MixMF, exclusive use of IF) and contextual level DHS survey decade (1990s, 2000s and 2010s) and economic factors (Gross Domestic Product by purchasing power parity, female wage and salaried workers, labor force participation rate female) as well as individual level sociodemographic (maternal age, maternal education, number of children in the household, wealth index, mother living with a partner, area of residence, mother working outside of home), and health factors (breastfed in the first hour, C-section).

**Results:**

Factors associated with EBF cessation were c-section (OR: 0.76; 95%CI: 0.64, 0.92), mothers working outside of the home (OR: 0.79; 95%CI: 0.69, 0.90), families in the highest income quintile (OR: 0.64; 95%CI: 0.49, 0.84), and female wage and salaried workers (OR: 0.92; 95%CI: 0.91, 0.94). MixMF was associated with women with higher education (OR: 1.54; 95%CI: 1.21, 1.97), mother working outside of the home (OR: 1.26; 95%CI: 1.10, 1.43), c-section (OR: 1.37; 95%CI: 1.15, 1.62), families in the highest income quintiles (OR: 2.77; 2.10, 3.65). and female wage and salaried workers (OR: 1.08;95% CI: 1.05, 1.09). Exclusive use of IF was associated with a mother working outside of the home (OR: 2.09; 95%CI: 1.41, 3.08), c-section (OR: 1.65; 95%CI: 1.09, 2.51), families in the highest income quintiles (OR: 12.08; 95% CI: 4.26, 34.28), the 2010s (OR: 3.81; 95%CI: 1.86, 7.79), and female wage and salaried workers (OR: 1.12; 95%CI: 1.07, 1.16).

**Discussion/Conclusion:**

Factors related to women empowerment and gender equality jeopardized EBF and favored the exclusive use of IF in Latin America. Therefore, workplace interventions to promote, protect, and support breastfeeding practices are key to reducing exclusive use of IF.

## Introduction

The global prevalence of exclusive breastfeeding (EBF) among infants under 6 months, as recommended by the World Health Organization, is 48%, which means significant progress is required to meet the Global Nutrition Goal of 70% by 2030 (1). However, the increased use of infant formula (IF) in low-and middle-income countries is concerning and has become a threat to achieving breastfeeding goals (2). A recent analysis indicated that countries could meet this goal by 2030, if national efforts to support breastfeeding practices and reduce the exclusive use of IF were scaled up (1). Therefore, understanding the modifiable factors associated with infant feeding practices can help countries protect, promote, and support breastfeeding (3).

Cessation of EBF before the 6 months of life, as recommended by the WHO, can occur due to sociodemographic, economic, and health factors (3). Teenage motherhood (4), primiparity (4), the lack of a skilled attendant at birth (5), and the absence of a partner (6) are associated with the early interruption of EBF (4–6). While breastfeeding in the first hour of life (BF1h) is a strong predictor for EBF (3, 7), cesarean delivery is associated with introducing milk-based prelacteal in hospitals (3, 8). Some studies have observed lower prevalence of EBF in urban areas compared to rural areas (9, 10), and one study found an increased prevalence of IF in urban areas (10). A meta-analysis of Brazilian studies noted that low education and low family income are also among the factors associated with EBF discontinuation before 6 months of age (4). In contrast, another study found that women with higher education contributed concomitantly to a significant increase in the prevalence of EBF and to the use of IF (2). Returning to work (11) and full-time work schedules are considered factors that contribute to the interruption of EBF (3, 12). Additionally, more than half of mothers who work outside the home offer food before 6 months of age (13). One study showed a negative correlation between Gross Domestic Product (GDP) and EBF and a positive correlation between GDP and breast-milk substitutes such as IF, meaning that IF use may become widespread as a country develops (14).

In Latin America and the Caribbean, the rate of EBF has increased slightly from 35% in 2005 to 38% in 2018, but at this annual growth rate, it would take more than 40 years to reach the 70% goal (15). A study of six Latin American and Caribbean countries that analyzed national surveys between the 1990s and the 2010s observed an increase in IF in infants under 6 months, especially among those living in urban areas (10). Likewise, a progressive increase in mixed milk feeding (MixMF, i.e., the provision of formula and/or animal milk along with breast milk) across all children’s age groups was observed (10). On the other hand, a progressive increase in EBF was observed, especially between the 1990s and 2000s and in the rural area. Findings from this study concluded that the increased use of IF has been a major barrier to achieving the 2030 Global Nutrition Goal for Exclusive Breastfeeding (10). However, prior studies in Latin America and the Caribbean have analyzed separately the factors associated with EBF or IF (4, 5, 16). In the present study, we aim to verify association between sociodemographic, economic, and health factors and EBF, MixMF, and the exclusive use of IF in three Latin American and Caribbean countries in the 1990s, 2000s, and 2010s.

## Methods

### Study design and data source

This cross-sectional time-series study used data from the Demographic and Health Surveys (DHS) Program conducted in the 1990s, 2000s, and 2010s. The DHS surveys are nationally representative household-based surveys that are comparable across and within countries at different time intervals. All DHS surveys used in this study are available on the DHS Program website^1^ (17) and were approved by each country’s ethics committee. The informed consent form was presented and signed by the respondents prior to the interview^2^ (18). In addition, we gathered data from the World Bank website, which is a publicly available website (19).

### Countries selection

The inclusion criteria used to choose countries in Latin America and the Caribbean were (1) the availability of at least one DHS survey for each decade (1990, 2000, and 2010), (2) the availability of variables on infant feeding on the day before the survey, and (3) the availability of variables on sociodemographic and health factors in the DHS survey databases. The Dominican Republic was not the only country considered eligible to this study because of sharp variation in the estimative of IF between datasets from the 2000s and 2010s, as our research group analyzed before (20).

The DHS Program website has available datasets for 15 countries in Latin America and the Caribbean. However, 11 countries did not have surveys for all decades (Brazil, Bolivia, Ecuador, El Salvador, Guatemala, Guyana, Honduras, Mexico, Nicaragua, Paraguay, and Trinidad and Tobago). The three countries with surveys in the three decades were Colombia, Haiti, and Peru. A total of 13 DHS surveys were included in this study: Colombia (4 databases: DHS III—1995, DHS IV—2000; DHS V—2005; DHS VI—2010), Haiti (5 databases: DHS III—1994/1995; DHS IV—2000; DHS V—2005/2006; DHS VI—2012; DHS VII—2016/2017), and Peru (4 databases: DHS III—1996; DHS IV—2000; DHS V—2007/2008; DHS VI—2012).

### DHS sample, target population, and analytical sampling

All DHS surveys feature complex two-stage probability sampling. In the first stage, the census sectors (conglomerates) were selected, and in the second stage, the households to survey were selected (21). In most DHS surveys, people eligible for the individual interview include women of reproductive age (15–49) with an infant under the age of three or five (22). The target population of this study consisted of infants under 6 months of age who were alive at the time of the interview and who lived with the respondent. Thus, the pooled analytical sample from the 13 DHS surveys included in this study consisted of 12,775 infants under 6 months of age. The percentage of infants excluded was 1.3% in Colombia and 4.6% in Haiti due to the exclusion of children older than 6 months, dead children, and those who did not live with the respondent, as recommended by the WHO (23, 24).

### Outcomes

This study selected three infant feeding outcomes defined by a recent WHO recommendation (22):

- *Exclusive breastfeeding* (EBF) is defined as the provision of only breast milk (numerator: infants under 6 months of age who were on EBF in the previous 24 h/denominator: infants 0–5 months old). Prescribed medicines, oral rehydration solution, vitamins, and minerals are not counted as fluids or foods (24).
- *Mixed milk feeding* (MixMF) is defined as the provision of formula and/or animal milk in addition to breast milk (numerator: infants 0–5 months of age who received breast milk, milk, and IF in the previous 24 h/denominator: infants 0–5 months old) (24).
- *Exclusive use of Infant formula* (exclusive use of IF) is defined as the exclusive use of IF to infants under 6 months old in the previous 24 h (numerator: infants younger than 6 months who exclusively received IF in the last 24 h/denominator: infants 0–5 months).

The outcomes were configured as dichotomous variables (no/yes). Missing data and the category “do not know” were less than 1% and were considered as “not consumed,” as recommended by the WHO (23, 24).

### Predictors

**Figure 1:**
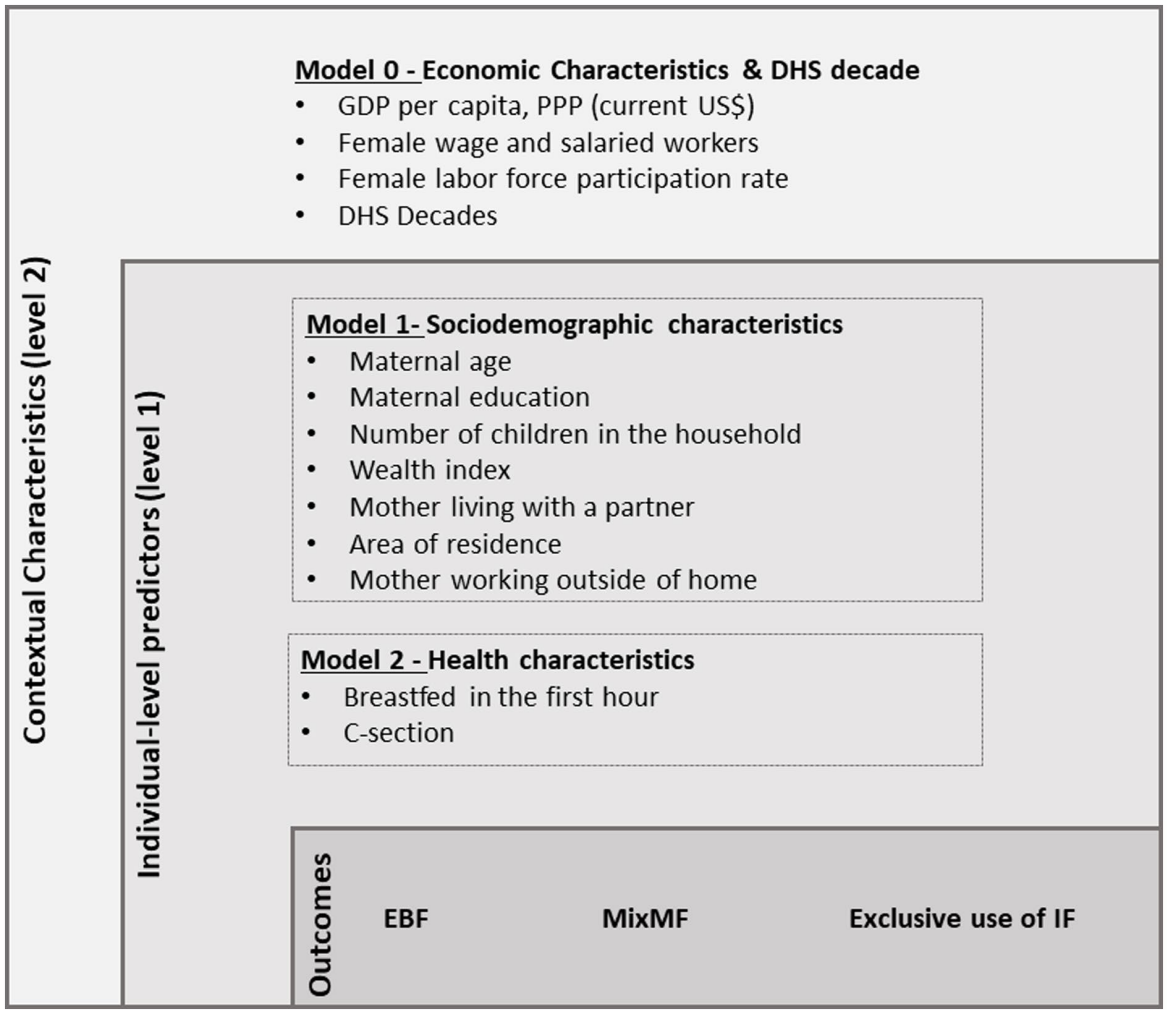
Analytical model of sociodemographic, health, and economic variables associated with exclusive breastfeeding (EBF), mixed milk feeding (MixMF), and exclusive use of infant formula (IF).

Sociodemographic, health, and economic predictors selection was guided by the hierarchical framework (25) developed based on models presented by previously studies supporting the associations with infant feeding (3, 26, 27) and according to the availability of information in the selected DHS surveys. These determinations informed the hierarchical framework illustrating potential associations of infant feeding used to guide our modeling approach (Figure 1).

Individual-level factors included sociodemographic and health obtained from the DHS surveys. The *sociodemographic factors* were maternal age (<20, 20–24 years, 25–29 years, ≥30 years), maternal education (no education, high school, and college education), number of children in the household (1, 2–3, and ≥ 4), wealth index (first quintile/s quintile/third quintile/fourth quintile/fifth quintile), mother living with a partner (no/yes), area of residence (urban/rural), mother working outside of the home (formal/informal) (no/yes). The wealth index refers to detailed information collected on the availability of consumer assets and housing characteristics that are directly related to the socioeconomic status (17). Each family received a score generated through a principal component data analysis to create the WI quintiles (17). The *health factors* were breastfeeding in the infant’s first hour of life (BF1h) (no/yes) and c-section (no/yes) (Figure 1).

Contextual level factors included economic variables obtained from the World Bank website and the DHS survey decade. The *economic factors* were gross domestic product by purchasing power parity (GDP PPP) (continuous variable), female wage and salaried workers (salaried workers/self-employed workers) (% of female employment), and female labor force participation rate (% of female population ages 15–64). The GDP PPP refers to the sum *per capita* in current international dollars of all final goods and services produced in a country in a given period. The female wage and salaried workers variable refers to the employment status of women that is distinguished into two categories of total employees: (a) salaried workers (also known as employees) and (b) self-employed workers. The female labor force participation rate variable refers to the proportion of the female population age 15 and over who provide labor for the production of goods and services and includes both employed and unemployed women. Both variables related to working women are estimates modeled by the International Labor Organization (ILO) (19). The *DHS survey decades* were grouped as follows: DHS surveys from 1990 to 1999 were grouped into the 1990s, DHS surveys from 2000 to 2009 into the 2000s, and DHS surveys from 2010 to 2019 into the 2010s.

### Data analysis

First, descriptive analysis of the outcome and predictors for each decade and each country was conducted individually (Supplementary Tables S1–S3). For these analyses, the complex sampling structure (stratification and conglomeration) was considered by weighting the importance and domain of the sample and the primary sampling unit using the *surveyset* command available in Stata.

Subsequently, the databases for each country and each decade were integrated (i.e., pooled database of countries). Descriptive analysis of the sociodemographic and health variables was conducted, including prevalence and respective confidence intervals of 95% by decades. Similarly, the average of the economic variables was calculated by decades for the pooled database of countries.

To verify the associations between the predictors and each outcome (EBF, MixMF, and exclusive use of IF), multilevel logistic regression models were conducted to estimate the odds ratios (OR) and 95% confidence intervals. For the multilevel logistic regression of the pooled database of countries, the weighting of strata, conglomerates, and sample weight was recoded to take into account the contextual level. For recoding the stratum, the cluster number, survey year, country code, and survey phase were considered. For recoding the conglomerate, the sample domain, country code, survey phase, and survey year were considered. For recoding the sample weight, the country code and survey phase were considered. The complex structure of the strata, conglomerates, and sample were incorporated into the multilevel analyses.

At this stage, economic and DHS survey decade factors comprised level 2 (contextual level), and sociodemographic and health factors comprised level 1 (individual level). A hierarchical approach was used to estimate the individual effect of the factors on the outcome (25), following a theoretical model established *a priori* (Figure 1). The hierarchical modeling data analysis approach (25) has been extensively used to study determinants of infant feeding practices (16, 27), as in the case of our analysis. First, the economic and DHS survey decade factors were added to the initial model (model 0), organizing individual data according to the clusters used in the multilevel analysis. Subsequently, in model 1, the sociodemographic factors were included; these factors were adjusted for in subsequent models. Likewise, factors related to health characteristics were added in model 2 to model 1 variables. At each level model, a value of *p* <0.05 was used as a statistical significance criterion to assess the correlation between factors and outcome. This hierarchical modeling process was repeated for each outcome individually. At the end of each model, the Intraclass Correlation Coefficient was calculated. All analyses were performed using STATA SE® version 15.1.

## Results

**Figure 2:**
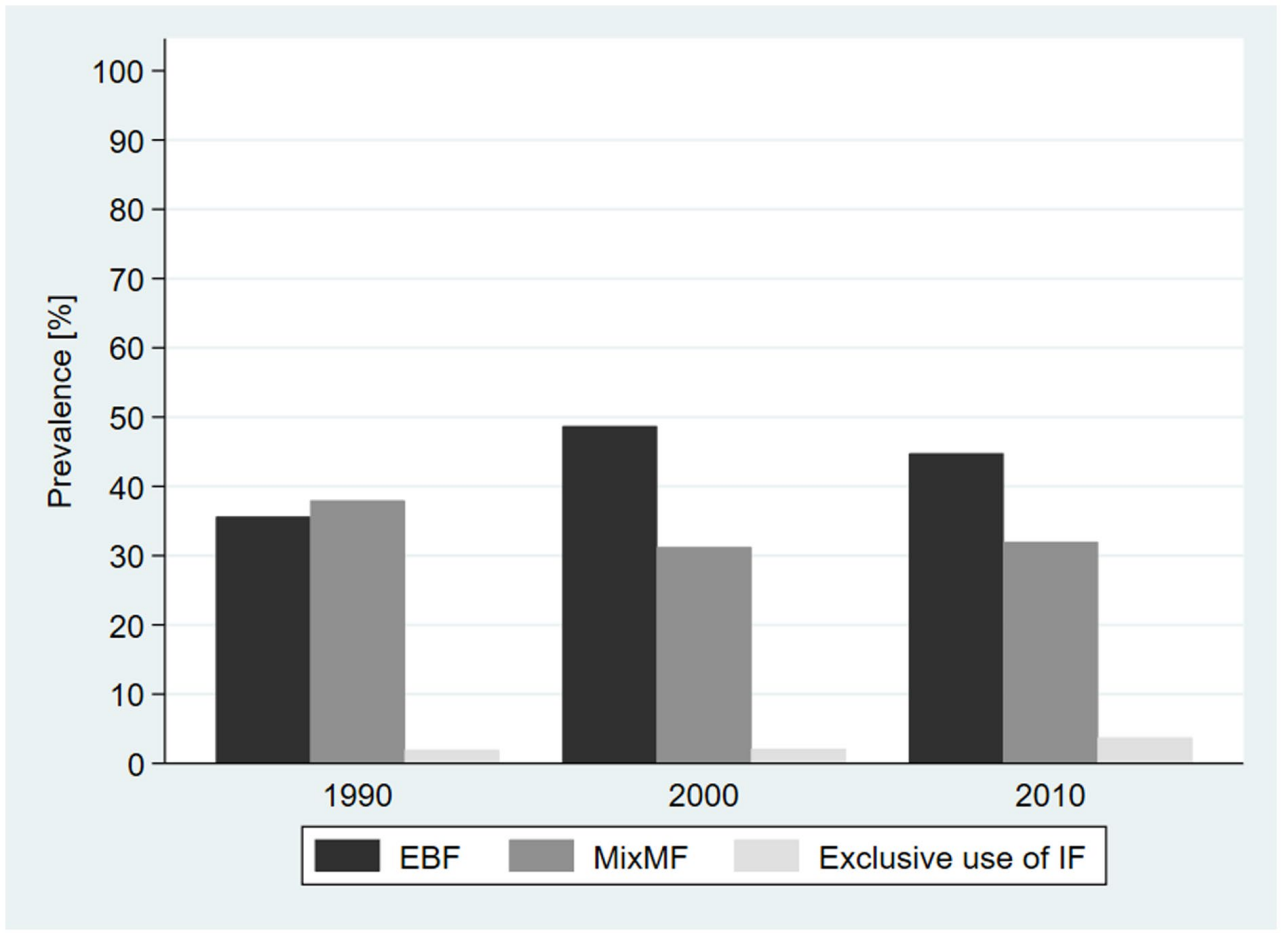
Prevalence of exclusive breastfeeding (EBF), mixed milk feeding (MixMF), and exclusive use of infant formula for the pool of countries by decade. DHS, 1990–2010.

The prevalence of EBF increased from 35.6% (95% CI = 33.1, 38.3) in 1990 to 44.8% (95% CI = 42.7, 46.9) in 2010. The prevalence of MixMF reduced from 37.9% (95% CI = 35.5, 40.4) in 1990 to 31.9% (95% CI = 30.1, 33.9) in 2010. The prevalence of exclusive use of IF increased from 1.9% (95% CI = 1.4, 2.8) in 1990 to 3.7% (95% CI = 2.9, 4.7) in 2010 (Figure 2).

In the pooled database of countries, no differences in the prevalence of outcomes were observed across area of residence and mothers’ age group across the decades. Between 1990 and 2010, the proportions of women with high school and college education increased in all three countries. The prevalence of women who reported working outside of the home, as well as those who reported living with a partner, reduced between 1990 and 2010. However, the prevalence of breastfeeding in the first hour and c-section increased across the decades. For the contextual level variables, the average number of female wage and salaried workers increased slightly from 30.3% in 1990 to 32.2% in 2010. The average female labor force participation increased from 55.6% in 1990 to 65.4% in 2010. The GDP PPP of the countries grew by US$2,610 between 1990 and 2010 (Table 1). The individual country surveys between 1990 and 2010 found an increase in maternal education, BF1h, c-section, the average female participation in the labor force, and the country GDP (Supplementary Tables S1–S3).

**Table 1:** Characterization of sociodemographic, health, and economic factors in the set of Latin American countries in the 1990s, 2000s, and 2010s.

Factors	1990 OR (95% CI)	2000 OR (95% CI)	2010 OR (95% CI)
GDP per capita, PPP	4313.1	6215.6	6923.1
Female wage and salaried workers	30.3	33.8	32.2
Female labor force participation rate	55.6	63.3	65.4
Maternal age			
<20	15.4 (13.8, 17.1)	16.6 (15.4, 17.8)	17.2 (15.7, 18.7)
20–24	28.6 (26.5, 30.8)	27.1 (25.6, 28.6)	27.7 (25.9, 29.5)
25–29	23.1 (21.2, 25.1)	22.6 (21.2, 24.1)	23.2 (21.6, 24.9)
≥30	32.9 (30.7, 35.3)	33.8 (32.1, 35.4)	31.9 (30.1, 33.9)
Maternal education			
No schooling	13.8 (12.3, 15.5)	9.7 (8.7, 15.5)	6.6 (5.7, 7.6)
Primary school	38.6 (36.2, 41.1)	35.5 (33.9, 37.1)	30.8 (29.1, 32.7)
High school	36.9 (34.5, 39.4)	41.4 (39.7, 43.2)	48.3 (46.3, 50.4)
College	10.7 (9.2, 12.4)	13.3 (12.1, 14.6)	14.2 (12.8, 15.8)
Number of children in the household			
1	32.3 (30.1, 34.5)	35.3 (33.7, 36.9)	37.3 (35.3, 39.2)
2–3	39.6 (37.4, 41.9)	41.1 (39.5, 42.8)	44.4 (42.4, 46.5)
≥4	28.1 (25.9, 30.3)	23.6 (22.2, 25.1)	18.3 (16.9, 19.8)
Mother living with a partner			
Yes	88.9 (87.4, 90.3)	82.3 (81.0, 83.6)	81.9 (80.3, 83.3)
No	11.1 (9.7, 12.5)	17.7 (16.4, 18.9)	18.1 (16.7, 19.7)
Residence area			
Urban	55.4 (52.7, 58.2)	55.9 (53.8, 57.9)	56.8 (54.1, 58.9)
Rural	44.6 (41.8, 47.3)	44.1 (42.1, 46.2)	43.2 (41.1, 45.2)
Mother working outside of home			
No	66.5 (64.1, 68.8)	64.7 (62.9, 66.4)	71.6 (69.7, 73.5)
Yes	33.5 (31.2, 35.9)	35.3 (33.6, 37.1)	28.4 (26.5, 30.3)
Breastfed in the first hour			
Yes	42.6 (40.1, 45.2)	53.2 (51.4, 54.9)	53.5 (51.5, 55.6)
No	57.4 (54.8, 59.9)	46.8 (45.1, 48.6)	46.5 (44.5, 48.5)
C-section			
No	89.3 (87.6, 90.8)	82.3 (80.8, 83.7)	76.2 (74.5, 77.9)
Yes	10.7 (9.2, 12.4)	17.7 (16.3, 19.2)	23.8 (22.1, 25.5)

EBF interruption was associated with mother working outside of the home (OR: 0.79; 95% CI: 0.69, 0.90), families in the fifth income quintile (OR: 0.64; 95% CI: 0.49, 0.84), c-section (OR: 0.76; 95% CI: 0.64, 0.92), and female wage and salaried workers (OR: 0.92; 95% CI: 0.91, 0.94). The continuation of EBF was associated with women living with a partner (OR: 1.38; 95% CI: 1.17, 1.65), and the prevalence was higher in the 2000s (OR: 1.66; 95% CI: 1.39, 1.98) (Table 2).

**Table 2:** Association of sociodemographic, health, and economic factors with exclusive breastfeeding (EBF) in three selected Latin American countries in the 1990s, 2000s, and 2010s.

Predictors	Exclusive breastfeeding (EBF)		
	Model 0 OR (95% CI)	Model 1 OR (95% CI)	Model 2 OR (95% CI)
Level 2—contextual predictors			
GDP per capita, PPP	1.00 (1.00, 1.00)		
Female wage and salaried workers	0.92 (0.91, 0.94)		
Female labor force participation rate	0.98 (0.97, 1.00)		
Decades			
1990	1.00 (ref)		
2000	1.66 (1.39, 1.98)		
2010	0.83 (0.68, 1.00)		
Level 1—individual-level predictors			
Model 1—sociodemographic characteristics			
Maternal age			
<20–24		1.00 (ref)	
25–29		1.07 (0.90, 1.26)	
≥30		0.99 (0.83, 1.19)	
Maternal education			
No schooling		1.00 (ref)	
High school		1.13 (0.98, 1.30)	
College		0.93 (0.72, 1.18)	
Number of children in the household			
1		1.00 (ref)	
2–3		1.12 (0.96, 1.30)	
≥4		1.10 (0.89, 1.38)	
Wealth index			
First quintile		1.00 (ref)	
Second quintile		1.02 (0.85, 1.23)	
Third quintile		0.89 (0.72, 1.10)	
Fourth quintile		0.79 (0.62, 1.00)	
Fifth quintile		0.64 (0.49, 0.84)	
Mother living with a partner			
No		1.00 (ref)	
Yes		1.38 (1.17, 1.65)	
Area of residence			
Urbana		1.00 (ref)	
Rural		1.09 (0.91, 1.32)	
Mother working outside of home			
No		1.00 (ref)	
Yes		0.79 (0.69, 0.90)	
Model 2—health characteristics			
Breastfed in the first hour			
No			1.00 (ref)
Yes			1.11 (0.98, 1.26)
C-section			
No			1.00 (ref)
Yes			0.76 (0.64, 0.92)
ICC	0.30	0.29	0.29

DHS, 1990–2010. Model 0: contextual predictors—level 2: Gross Domestic Product (GDP) per capita, purchasing power parity (PPP); female wage and salaried workers; female labor force participation rate; decades. Model 1: Individual-level predictors—Level 1: sociodemographic characteristics (maternal age, maternal education, number of children in the household, wealth index, mother living with a partner, area of residence, mother working outside of home) adjusted for all contextual variables. Model 2: Individual-level predictors—Level 1, sociodemographic characteristics and health characteristics (breastfed in the first hour, c-section) adjusted for all contextual variables. ICC, Intraclass Correlation Coefficient.

MixMF was associated with women with a college education (OR: 1.54; 95% CI: 1.21, 1.97), families in the highest income quintiles (OR: 2.77; 95% CI: 2.10, 3.65), working outside of the home (OR: 1.26; 95% CI: 1.10, 1.43), c-section (OR: 1.37; 95% CI: 1.15, 1.62), and wage and salaried workers (OR: 1.08; 95% CI: 1.05, 1.09). Living with a partner (OR: 0.65; 95% CI: 0.54, 0.79), residing in rural areas (OR: 0.82; 95% CI: 0.68, 0.98), breastfeeding in the first hour (OR: 1.37; 95% CI: 1.15, 1.62), and the 2000s (OR: 0.65; 95% CI: 0.54, 0.77) reduced the odds of MixMF (Table 3).

**Table 3:** Association of sociodemographic, health, and economic factors with breast milk supplemented with mixed milk feeding (MixMF) in the three selected Latin American countries in the 1990s, 2000s, and 2010s.

Predictors	Mixed milk feeding (MixMF)		
	Model 0 OR (95% CI)	Model 1 OR (95% CI)	Model 2 OR (95% CI)
Level 2—contextual predictors			
GDP per capita, PPP	1.00 (1.0, 1.0)		
Female wage and salaried workers	1.08 (1.05, 1.09)		
Female labor force participation rate	1.01 (0.99, 1.02)		
Decades			
1990	1.00 (ref)		
2000	0.65 (0.54, 0.77)		
2010	0.97 (0.79, 1.18)		
Level 1—individual-level predictors			
Model 1 sociodemographic characteristics			
Maternal age			
<20–24		1.00 (ref)	
25–29		1.11 (0.94, 1.30)	
≥30		1.15 (0.95, 1.38)	
Maternal education			
No schooling		1.00 (ref)	
High school		1.09 (0.92, 1.29)	
College		1.54 (1.21, 1.97)	
Number of children in the household			
1		1.00 (ref)	
2–3		0.92 (0.79, 1.07)	
≥4		1.01 (0.81, 1.27)	
Wealth index			
First quintile		1.00 (ref)	
Second quintile		1.28 (1.03, 1.57)	
Third quintile		1.51 (1.21, 1.88)	
Fourth quintile		1.73 (1.35, 2.21)	
Fifth quintile		2.77 (2.10, 3.65)	
Mother living with a partner			
No		1.00 (ref)	
Yes		0.65 (0.54, 0.79)	
Area of residence			
Urbana		1.00 (ref)	
Rural		0.82 (0.68, 0.98)	
Mother working outside of the home			
No		1.00 (ref)	
Yes		1.26 (1.10, 1.43)	
Model 2—health characteristics			
Breastfed in the first hour			
No			1.00 (ref)
Yes			0.87 (0.76, 0.98)
C-section			
No			1.00 (ref)
Yes			1.37 (1.15, 1.62)
ICC	0.27	0.21	0.21

DHS, 1990–2010. Model 0: contextual predictors—level 2: Gross Domestic Product (GDP) per capita, purchasing power parity (PPP); female wage and salaried workers; female labor force participation rate; decades. Model 1: Individual-level predictors—Level 1: sociodemographic characteristics (maternal age, maternal education, number of children in the household, wealth index, mother living with a partner, area of residence, mother working outside of home) adjusted for all contextual variables. Model 2: Individual-level predictors—Level 1: sociodemographic characteristics and health characteristics (breastfed in the first hour, c-section) adjusted for all contextual variables. ICC, Intraclass Correlation Coefficient.

Exclusive use of IF was associated with families in the highest income quintiles (OR: 12.08; 95% CI: 4.26, 34.28), working outside of the home (OR: 2.09; 95% CI: 1.41, 3.08), c-section (OR: 1.65; 95% CI: 1.09, 2.51), wage and salaried workers (OR: 1.12; 95% CI: 1.07, 1.16), and the 2010s (OR: 3.81; 95% CI: 1.86, 7.79). Having two or more children (OR: 0.57; 95% CI: 0.37, 0.88) and breastfeeding in the first hour (OR: 0.66; 95% CI: 0.46, 0.97) reduced the odds of exclusive IF use (Table 4).

**Table 4:** Association of sociodemographic, health, and economic factors with exclusive use of infant formula (IF) in the set of Latin American countries in the 1990s, 2000s, and 2010s.

Predictors	Exclusive use of infant formula (IF)		
	Model 0 OR (95% CI)	Model 1 OR (95% CI)	Model 2 OR (95% CI)
Level 2—contextual predictors			
GDP per capita, PPP	0.99 (0.99, 0.99)		
Female wage and salaried workers	1.12 (1.07, 1.16)		
Female labor force participation rate	1.00 (0.94, 1.05)		
Decades			
1990	1.00 (ref)		
2000	1.02 (0.59, 1.74)		
2010	3.81 (1.86, 7.79)		
Level 1—individual-level predictors			
Model 1—sociodemographic characteristics			
Maternal age			
<20–24		1.00 (ref)	
25–29		1.19 (0.76, 1.87)	
≥30		1.37 (0.80, 2.32)	
Maternal education			
No schooling		1.00 (ref)	
High school		1.03 (0.64, 1.68)	
College		0.92 (0.50, 1.72)	
Number of children in the household			
1		1.00 (ref)	
2–3		0.57 (0.37, 0.88)	
≥ 4		0.41 (0.21, 0.81)	
Wealth index			
First quintile		1.00 (ref)	
Second quintile		6.31 (2.40, 16.63)	
Third quintile		8.91 (3.45, 22.99)	
Fourth quintile		6.38 (2.31, 17.62)	
Fifth quintile		12.08 (4.26, 34.28)	
Mother living with a partner			
No		1.00 (ref)	
Yes		1.15 (0.74, 1.78)	
Area of residence			
Urbana		1.00 (ref)	
Rural		0.56 (0.32, 0.98)	
Mother working outside of the home			
No		1.00 (ref)	
Yes		2.09 (1.41, 3.08)	
Model 2—health characteristics			
Breastfed in the first hour			
No			1.00 (ref)
Yes			0.66 (0.46, 0.97)
C-section			
No			1.00 (ref)
Yes			1.65 (1.09, 2.51)
ICC	0.48	0.44	0.45

DHS, 1990–2010. Model 0: contextual predictors—level 2: Gross Domestic Product (GDP) per capita, purchasing power parity (PPP); female wage and salaried workers; female labor force participation rate; decades. Model 1: Individual-level predictors—Level 1: sociodemographic characteristics (maternal age, maternal education, number of children in the household, wealth index, mother living with a partner, area of residence, mother working outside of home) adjusted for all contextual variables. Model 2: Individual-level predictors—Level 1: sociodemographic characteristics and health characteristics (breastfed in the first hour, c-section) adjusted for all contextual variables. ICC, Intraclass Correlation Coefficient.

Through the Intraclass Correlation Coefficient, we identified that 29% of the increase in EBF, 21% of the increase in MixMF, and 45% of the increase in exclusive IF use in this period were attributed to contextual factors (Tables 2–4).

## Discussion

Our study is the first, as far as we know, to explore the individual and contextual factors associated with EBF, MixMF, and exclusive use of IF in three countries in Latin America and the Caribbean. The prevalence of EBF improved, with the largest increase seen between 1990 and 2000, followed by a stabilization of the rate between 2000 and 2010. These data corroborate information compiled in the Global Breastfeeding Scorecard, which indicated a worldwide increase of 10 % in EBF between 2010 and 2022, reaching 48% (1). However, such progress is uneven and insufficient (1). To achieve the 70% breastfeeding target set as a 2030 global goal, modifiable factors must be identified, disparities reduced, and equity promoted for women who need support to breastfeed.

Our study explored the association of sociodemographic, economic, and health factors with the indicators EBF, MixMF, and exclusive use of IF between the 1990s and 2010s. In the 1990s and 2000s, an increase in the prevalence of EBF was observed, while MixMF decreased and exclusive use of IF increased between 1990 and 2010. For women who were employed, who delivered by cesarean section, and who were classified in the highest income quintile, EBF decreased; however, for women who had a partner and in the 2000s, EBF increased. For women with a partner, who resided in rural areas and in the 2000s, MixMF reduced; while for women with a college education, this indicator increased. For women with greater numbers of children, who practiced breastfeeding in the first hour, and in the 2010s, exclusive use of IF consumption reduced. For women who were employed, who delivered by c-section, and who were in higher income quintiles, MixMF and exclusive use of IF consumption increased.

We found in our study that the likelihood of EBF increased when women living with a partner. These findings corroborate a Brazilian study that observed that not living with a partner was negatively associated with the prevalence of EBF (6). On the one hand, we found that EBF reduces with cesarean birth, indicating agreement with some studies that showed that c-section could increase the risk of pre-lacteal supplementation (8), and distribution of free samples of breast-milk substitutes in the hospital environment (3) hinders maintenance of EBF. On the other hand, vaginal delivery contributes to timely initiation of breastfeeding and facilitates the maintenance of EBF (3, 8). Some variables contributed to the negative outcome of breastfeeding, thus highlighting the importance of the family and community support network, breastfeeding counseling, and training health professionals to have adequate skills to provide training, practical knowledge, and confidence to women during prenatal and postpartum care.

The variables maternal work and formal or informal employment also showed negative outcomes in relation to EBF, corroborating two studies that showed that employed mothers may interrupt EBF early (13). The employment situation of mothers should be analyzed, including mothers who do not work outside the home and mothers who work outside the home but may or may not take maternity leave because the absence of maternity leave is also associated with the interruption of EBF (28). The maternal employment variable should be investigated considering whether or not the mother is on maternity leave because employed mothers on maternity leave probably have better conditions for maintaining EBF during the maternity leave period. Six-month maternity leave, a supportive work environment, and flexible work schedules are contextual factors that contribute to EBF up to 6 months of age (3, 12). Countries also need to be concerned with providing equity and reducing disparities in relation to the labor market for breastfeeding women because not all countries have laws that provide six-month paid maternity leave after the birth of the child and legislate the mandatory provision of paid breaks and support facilities for breastfeeding after returning to work. Protecting maternity in the workplace and promoting family and workplace leave policies are important indicators for successful breastfeeding (29).

Contextual factors, including income classification, are part of the determinants of breastfeeding (3). In this study, the prevalence of EBF also decreased when women were classified in the highest income quintile of the wealth index. Our study collaborates with other studies, which reported that mothers with higher income might breastfeed for less time (3, 30). This trend could be due to the misperception that breastfeeding is for the poor and unsophisticated and that breast-milk substitutes are modern and prestigious (3). In addition to national efforts to achieve breastfeeding goals, it would also be interesting to provide equity and quality counseling on the benefits of breastfeeding to women who are more likely to discontinue breastfeeding.

Although the prevalence of exclusive use of IF between the decades has increased, the prevalence is still low. Feeding a child exclusively with IF can be very expensive and almost unfeasible for infants older than 3 months when caregivers normally introduce other types of milk or foods for the infant. Perhaps this is one of the reasons why we found such a low prevalence of exclusive use of IF in this population. In previous studies (2, 14), the prevalence of IF was analyzed together with breastfeeding (MixMF), but the exclusive consumption of IF was not analyzed. Therefore, this study advances the understanding of factors associated with exclusive use of IF. The increase in economic development in low-and middle-income countries, the enrichment of the population, and the continuation of social changes unfavorable to breastfeeding could lead to increased commercialization of IF, as evidence shows that IF is a product that is resistant to market crises (3, 14). One possible factor currently contributing to the increase in IF consumption that was not examined in this study is the aggressive marketing of IF. Evidence is powerful of the connection between marketing of breast-milk substitutes and the decision of families with infants and toddlers about breastfeeding and infant health (31). To enhance efforts by countries to reduce IF consumption and increase breastfeeding rates, trends in breastfeeding and infant feeding indicators should be analyzed (31). Data from 2022 showed that the milk powder industry used systematic and unethical marketing strategies to influence families’ infant feeding decisions (31), which may justify the progressive increase in IF consumption rates.

Regarding health-associated factors, one study noted that cesarean section was associated with a higher chance of introducing milk-based prelacteals in hospitals (8), and our study also noted that exclusive use of IF increased for women who had c-sections. The promotion and availability of IF through the marketing of breast-milk substitute industries influence the early introduction of foods in infants under 6 months of age (3). Factors associated with the organization of services and support network are also important, including the unpreparedness of the health professional team and the lack of organization of health services to assist the mother in breastfeeding (3, 12). Although these are important factors, they were not measured in the DHS surveys.

Exclusive use of IF also increased for women ranked in higher income quintiles. Furthermore, when adjusted for contextual variables, the 2010s appear to have contributed to the increase in exclusive use of IF, i.e., more than 44% of the increase in exclusive use of IF in this period was concentrated in the contextual factors in the countries studied. We emphasize that such results align with the findings that IF consumption in the first 8 months of life increased in Latin America and the Caribbean, the Middle East and North Africa, Eastern Europe, and Central Asia (14). Exclusive use of IF also increased for those women who were employed and is one of the main reasons for not breastfeeding or weaning early and facilitates the introduction of other foods for infants under 6 months (3).

Moreover, we observed that exclusive use of IF reduced for women with more children and when they did BF1h. Collaborating with our study, researchers have observed that primiparity influences the interruption of EBF (4) and that breastfeeding in the first hour facilitates EBF until the sixth month (3, 7). A recent analysis observed that more than half of parents and pregnant women were exposed to aggressive marketing for formula milk, according to the WHO and UNICEF (31). To meet the challenges of increasing EBF and reducing IF consumption, governments, healthcare professionals, and the baby food industry need to stop the abusive marketing of milk formula and must fully implement and comply with the requirements of the International Code of Marketing of Breast-milk Substitutes (31). This requires passing, monitoring, and enforcing laws to prevent the promotion of milk formula in accordance with the International Code; investing in breastfeeding policies and programs; adequately funding paid parental leave; ensuring adequate quality support for breastfeeding; requiring the industry to publicly commit to full compliance with the Code and the 2023 World Health Assembly global decisions; and prohibiting health professionals from accepting sponsorship, scholarships, awards, grants, meetings, or events from companies that market foods for infants and young children (31).

Our study has some limitations to consider when interpreting the results. The first limitation is the differences in the number of food variables by survey year in the DHS surveys. In 2008, the WHO systematized the child nutrition indicators for Latin America and the Caribbean for the number of food variables for the surveys in these countries. Therefore, this increase could make the prevalence of EBF lower in the later years of the surveys compared to surveys in previous years, as it gives the mother more feeding options to remember. However, we found an increase in EBF from 1990 to 2010 and stabilization between 2000 and 2010, suggesting that potential misclassification was irrelevant. The lack of data in the DHS surveys regarding the age at which food was introduced is a limitation; therefore, we only estimated whether or not the infant consumed selected foods in the study. A limitation was the lack of information on maternity leave in the DHS surveys and the World Bank database since maternity leave facilitates the maintenance of EBF. Another limitation was the lack of more recent surveys for Colombia and Peru, as the most recent surveys for these countries were done more than 10 years ago. A DHS survey for Colombia in 2015 was available but does not have child-feeding data, and an ENDES survey for Peru in 2018 was available but did not contain sociodemographic and health data, so they were not included in our dataset. We checked the UNICEF website (32) and searched country websites, but no recent infant feeding data exists. Another limitation is the absence of infant feeding data along with sociodemographic and health data from Latin American countries, such as Brazil and Mexico with large populations of children under 2 years of age. These countries are important markets for the breast-milk substitutes and infant formula industries.

Despite these limitations, the strengths of our study are the use of nationally representative surveys, the analysis of three Latin American countries for three decades, and the association analysis of infant feeding indicators with sociodemographic, health, and economic factors to understand which factors impact EBF for infant up to 6 months of age as well as the supply of breast-milk substitutes and IF. Moreover, we used robust multilevel regression analysis for the pool of countries with recoding of the weighting aimed at reflecting the pool of countries and adjusting for the contextual level of the model. Thus, our analyses are important to support health professionals and especially health managers to understand the situation of infant feeding in the first 6 months of life in Latin America and the Caribbean, as national efforts are needed to increase rates of EBF until the sixth month and reduce rates of breast-milk substitutes and IF.

We concluded that the prevalence of EBF and exclusive use of IF both increased, while the prevalence of MixMF decreased in the studied decades. On the one hand, the cesarean delivery route and employed women with higher income exhibited a negative correlation with EBF. On the other hand, women living with a partner and the 2000s positively influenced EBF. Women who breastfed in the first hour, who had a partner, and who resided in rural areas, as well as the 2000s showed a negative correlation to MixMF. In contrast, women with a college education and wage and salaried workers influenced the increase of this indicator. Women who practiced breastfeeding in the first hour, who had 2–3 children, and ≥ 4 children were negatively associated with exclusive use of IF. Nevertheless, the 2010s and women with higher female labor force participation rate influenced the increase of this indicator. Both MixMF and exclusive use of IF increased among women who worked outside of the home, children born via c-section, and families ranked in the highest income quintiles. We also noted that the increases in EBF and MixMF were partly attributed to the 2000s, and the increase in exclusive IF use to the 2010s. We highlight the low prevalence of exclusive IF consumption for the pool of countries over the three decades because we do not frequently observe similar data in the literature.

The individual and contextual variables analyzed in this study captured the important influence of the political, economic, and social context on breastfeeding. This reinforces that breastfeeding does not have just one individual component but involves several components, including individual, contextual, and health. Hence, our analyses contribute to understanding factors that facilitate or impede the achievement of the Global Nutrition Goals for breastfeeding since it is not only about achieving breastfeeding prevalence rates, but also about equity for women who need support to breastfeed and reducing disparities in their environment.

## Data availability statement

Publicly available datasets were analyzed in this study. This data can be found at: The DHS and MICS are free access and available at: https://dhsprogram.com/data/available-datasets.cfm and https://mics.unicef.org/surveys.

## Author contributions

CM contributed to the conception and design of the study, data analysis, interpretation of results, writing of the manuscript, and approval of the final version. GB contributed to the data analysis, interpretation of results, writing of the manuscript, and approval of the final version. CA contributed to writing of the manuscript and approval of the final version. WC contributed to the conception and design of the study and approval of the final version. AR contributed to the conception and design of the study, data analysis, interpretation of results, writing of the manuscript, and approval of the final version. All authors contributed to the article and approved the submitted version.

## Footnotes

^1^https://dhsprogram.com/

^2^https://dhsprogram.com/Methodology/Protecting-the-Privacy-of-DHS-Survey-Respondents.cfm
