# Supplementary material for: Infant feeding practices in three Latin American countries in three decades: what demographic, health, and economic factors are relevant?
Source: Front Nutr. 2023 Oct 4;10:1239503. doi: 10.3389/fnut.2023.1239503 (PMC10582640; doi:10.3389/fnut.2023.1239503)
Supplement: Supplementary file 1 [file Table_1.docx]

Supplementary Material

**Infant feeding practices in three Latin American countries in three decades: what demographic, health, and economic factors are relevant?**

Camila Abadia Rodrigues Meira^1*^, Gabriela Buccini^2^, Catarina Machado Azeredo^1^, Wolney Lisbôa Conde ^3^, Ana Elisa Madalena Rinaldi^1^

*** Correspondence:** Corresponding Author: [camila_abadia8@hotmail.com](mailto:camila_abadia8@hotmail.com)

**Supplementary table 1:** Characterization of sociodemographic, health and economic factors in Colombia according to the research decade. DHS, 1990-2010.

| **Colombia** | **1990** |  | **2000** | **2010** |
| --- | --- | --- | --- | --- |
| **GDP per capita, PPP*** | 6402.0 |  | 7968.3 | 10732.0 |
| **Female wage and salaried workers** | 49.0 |  | 51.0 | 48.0 |
| **Female labor force participation rate** | 56.0 |  | 56.2 | 60.0 |
| **Maternal age** |  |  |  |  |
| < 20 | 18.8(15.4,22.6) |  | 20.8(18.6,23.1) | 22.8(20.2,25.7) |
| 20-24 | 30.9(26.8,35.3) |  | 29.2(26.7,31.9) | 29.5(26.8,32.5) |
| 25-29 | 26.9(23.1,31.1) |  | 21.8(19.5,24.3) | 22.3(19.7,25.1) |
| ≥ 30 | 23.4(19.8,27.5) |  | 28.2(25.6,31.0) | 25.4(22.6,28.3) |
| **Maternal Education** |  |  |  |  |
| No schooling | 5.2(3.6,7.5) |  | 3.5(2.6,4.6) | 1.8(1.2,2.8) |
| Primary school | 38.9(34.5,43.5) |  | 29.6(27.1,32.3) | 20.9(18.5,23.6) |
| High school | 48.8(44.0,53.5) |  | 54.0(510,56.9) | 58.6(55.5,61.7) |
| College | 7.1(5.0,10.0) |  | 12.9(10.9,15.2) | 18.7(16.2,21.5) |
| **Number of children in the household** |  |  |  |  |
| 1 | 39.3(35.2,43.5) |  | 37.1(34.4,39.9) | 41.5(38.3,44.7) |
| 2-3 | 42.2(38.1,46.3) |  | 46.5(43.6,49.4) | 45.7(42.5,48.9) |
| ≥ 4 | 18.5(15.3,22.3) |  | 16.4(14.2,18.8) | 12.8(10.9,15.0) |
| **Wealth index** |  |  |  |  |
| 1st quintile | 20.2(16.3,24.7) |  | 18.4(16.1,21.1) | 11.2(9.4,13.4) |
| 2st quintile | 20.1(16.5,24.1) |  | 16.7(14.7,18.9) | 16.2(13.9,18.7) |
| 3st quintile | 19.6(16.2,23.5) |  | 17.9(15.8,20.3) | 16.2(13.9,18.6) |
| 4st quintile | 19.8(16.3,23.7) |  | 21.8(19.3,24.5) | 23.8(21.1,26.7) |
| 5st quintile | 20.4(16.8,24.5) |  | 25.1(22.4,28.2) | 32.6(29.5,35.9) |
| **Mother living with a partner** |  |  |  |  |
| Não | 18.5(15.4,22.2) |  | 25.1(22.7,27.6) | 25.3(22.6,28.3) |
| Sim | 81.5(77.8,84.6) |  | 74.9(72.4,77.3) | 74.7(71.7,77.4) |
| **Area of residence** |  |  |  |  |
| Urbana | 63.0(57.6,68.1) |  | 68.6(65.5,71.5) | 73.2(69.9,76.1) |
| Rural | 36.9(31.9,42.4) |  | 31.4(28.5,34.5) | 26.8(23.9,30.0) |
| **Mother working outside of home** |  |  |  |  |
| Não | 67.0(62.7,71.2) |  | 68.7(65.8,71.5) | 72.0(69.0,74.8) |
| Sim | 32.9(28.8,37.3) |  | 31.3(28.5,34.2) | 27.9(25.2,30.9) |
| **Breastfed in the first hour** |  |  |  |  |
| Não | 55.5(51.1,59.8) |  | 41.2(38.4,44.1) | 40.9(37.8,44.2) |
| Sim | 44.5(40.2,48.9) |  | 58.8(55.9,61.6) | 59.1(55.8,62.2) |
| **C-section** |  |  |  |  |
| Não | 81.0(77.4,84.2) |  | 71.2(68.4,73.8) | 61.2(58.1,64.3) |
| Sim | 18.9(15.8,22.6) |  | 28.8(26.2,31.6) | 38.8(35.7,41.9) |
