# Supplementary material for: Infant feeding practices in three Latin American countries in three decades: what demographic, health, and economic factors are relevant?
Source: Front Nutr. 2023 Oct 4;10:1239503. doi: 10.3389/fnut.2023.1239503 (PMC10582640; doi:10.3389/fnut.2023.1239503)
Supplement: Supplementary file 2 [file Table_2.docx]

Supplementary Material

**Infant feeding practices in three Latin American countries in three decades: what demographic, health, and economic factors are relevant?**

Camila Abadia Rodrigues Meira^1*^, Gabriela Buccini^2^, Catarina Machado Azeredo^1^, Wolney Lisbôa Conde ^3^, Ana Elisa Madalena Rinaldi^1^

*** Correspondence:** Corresponding Author: [camila_abadia8@hotmail.com](mailto:camila_abadia8@hotmail.com)

**Supplementary table 2:** Characterization of sociodemographic, health and economic factors in Haiti according to the research decade. DHS, 1990-2010.

| **Haiti** | **1990** |  | **2000** | **2010** |
| --- | --- | --- | --- | --- |
| **GDP per capita, PPP*** | 1252.0 |  | 1438.0 | 1674.3 |
| **Female wage and salaried workers** | 13.0 |  | 15.1 | 17.5 |
| **Female labor force participation rate** | 59.0 |  | 59.1 | 61.9 |
| **Maternal age** |  |  |  |  |
| < 20 | 9.9(6.7,14.4) |  | 15.5(13.3,18.1) | 13.5(11.5,15.7) |
| 20-24 | 24.9(20.3,30.0) |  | 25.5(22.3,29.1) | 26.5(23.6,29.5) |
| 25-29 | 24.7(20.5,29.5) |  | 22.5(19.3,26.1) | 25.3(22.7,28.0) |
| ≥ 30 | 40.5(35.3,45.8) |  | 36.4(33.0,39.9) | 34.8(31.9,37.8) |
| **Maternal Education** |  |  |  |  |
| No schooling | 44.4(37.9,51.1) |  | 29.9(25.8,34.3) | 13.8(11.8,16.2) |
| Primary school | 41.1(34.9,47.6) |  | 47.3(43.3,51.2) | 43.3(40.0,46.7) |
| High school | 14.3(10.6,19.0) |  | 21.9(18.3,26.1) | 37.6(34.4,41.0) |
| College | 0.2(0.0,1.5) |  | 0.9(0.4,1.7) | 5.2(3.7,7.2) |
| **Number of children in the household** |  |  |  |  |
| 1 | 24.2(19.9,29.1) |  | 32.8(29.4,36.3) | 34.3(31.4,37.2) |
| 2-3 | 38.1(32.9,43.6) |  | 35.5(32.2,38.9) | 42.9(39.7,46.2) |
| ≥ 4 | 37.7(31.7,44.1) |  | 31.7(28.0,35.6) | 22.8(20.4,25.5) |
| **Wealth index** |  |  |  |  |
| 1st quintile | 22.4(16.5,29.8) |  | 17.4(13.9,21.4) | 15.8(13.4,18.6) |
| 2st quintile | 19.9(15.9,24.8) |  | 16.8(13.8,20.3) | 17.7(15.1,20.5) |
| 3st quintile | 20.3(16.1,25.3) |  | 20.9(16.5,26.0) | 18.5(15.9,21.4) |
| 4st quintile | 18.6(13.7,24.8) |  | 21.7(17.9,25.9) | 21.8(19.1,24.8) |
| 5st quintile | 18.7(13.7,24.9) |  | 23.3(18.5,28.9) | 26.2(22.9,29.8) |
| **Mother living with a partner** |  |  |  |  |
| Não | 9.7(8.8,10.7) |  | 16.9(15.9,18.0) | 15.9(14.7,17.3) |
| Sim | 90.3(89.3,91.2) |  | 83.1(81.9,84.1) | 84.0(82.7,85.3) |
| **Area of residence** |  |  |  |  |
| Urbana | 48.8(46.4,51.2) |  | 56.5(54.7,58.4) | 50.7(48.9,52.4) |
| Rural | 51.2(48.8,53.6) |  | 43.5(41.6,45.3) | 49.3(47.6,51.1) |
| **Mother working outside of home** |  |  |  |  |
| Não | 69.3(63.2,74.7) |  | 66.8(62.8,70.7) | 74.2(71.0,77.2) |
| Sim | 30.7(25.3,36.8) |  | 33.2(29.3,37.2) | 25.8(22.8,28.9) |
| **Breastfed in the first hour** |  |  |  |  |
| Não | 59.6(57.9,61.3) |  | 45.4(43.9,46.9) | 44.8(43.1,46.6) |
| Sim | 40.4(38.7,42.1) |  | 54.6(53.1,56.1) | 55.2(53.4,56.9) |
| **C-section** |  |  |  |  |
| Não | 98.7(96.9,99.5) |  | 97.2(95.7,98.1) | 94.3(92.7,95.6) |
| Sim | 1.3(0.5,3.1) |  | 2.8(1.9,4.3) | 5.7(4.4,7.3) |
