# Supplementary material for: Infant feeding practices in three Latin American countries in three decades: what demographic, health, and economic factors are relevant?
Source: Front Nutr. 2023 Oct 4;10:1239503. doi: 10.3389/fnut.2023.1239503 (PMC10582640; doi:10.3389/fnut.2023.1239503)
Supplement: Supplementary file 3 [file Table_3.docx]

Supplementary Material

**Infant feeding practices in three Latin American countries in three decades: what demographic, health, and economic factors are relevant?**

Camila Abadia Rodrigues Meira^1*^, Gabriela Buccini^2^, Catarina Machado Azeredo^1^, Wolney Lisbôa Conde ^3^, Ana Elisa Madalena Rinaldi^1^

*** Correspondence:** Corresponding Author: [camila_abadia8@hotmail.com](mailto:camila_abadia8@hotmail.com)

**Supplementary table 3:** Characterization of sociodemographic, health and economic factors in Peru according to the research decade. DHS, 1990-2010

| **Peru** | **1990** |  | **2000** | **2010** |
| --- | --- | --- | --- | --- |
| **GDP per capita, PPP*** | 4570.0 |  | 7289.1 | 10768.0 |
| **Female wage and salaried workers** | 31.0 |  | 35.4 | 39.0 |
| **Female labor force participation rate** | 55.0 |  | 66.4 | 74.0 |
| **Maternal age** |  |  |  |  |
| < 20 | 15.4(13.4,17.8) |  | 13.9(12.4,15.6) | 13.1(10.5,16.2) |
| 20-24 | 28.6(25.8,31.6) |  | 26.1(23.9,28.4) | 26.3(22.7,30.3) |
| 25-29 | 21.3(17.9,25.1) |  | 23.3(21.3,25.4) | 21.3(17.9,25.1) |
| ≥ 30 | 34.6(31.6,37.8) |  | 36.7(34.2,39.2) | 39.3(35.1,43.8) |
| **Maternal Education** |  |  |  |  |
| No schooling | 9.5(7.8,11.4) |  | 5.0(4.1,6.1) | 2.9(1.9,4.4) |
| Primary school | 37.9(34.6,41.3) |  | 34.5(32.2,36.9) | 27.3(23.6,31.3) |
| High school | 38.0(34.6,41.6) |  | 41.0(38.5,43.6) | 47.9(43.2,52.6) |
| College | 14.6(12.3,17.2) |  | 19.4(17.4,21.7) | 21.9(18.2,26.2) |
| **Number of children in the household** |  |  |  |  |
| 1 | 31.7(28.8,34.8) |  | 35.0(32.6,37.5) | 34.6(30.7,38.8) |
| 2-3 | 39.1(35.9,42.3) |  | 39.7(37.3,42.2) | 44.9(40.9,48.9) |
| ≥ 4 | 29.2(26.4,32.2) |  | 25.3(23.2,27.4) | 20.5(17.4,24.0) |
| **Wealth index** |  |  |  |  |
| 1st quintile | 15.1(12.8,17.7) |  | 16.2(14.5,18.1) | 16.6(13.5,20.1) |
| 2st quintile | 17.6(15.2,20.3) |  | 16.9(15.2,18.8) | 18.5(15.5,22.0) |
| 3st quintile | 20.7(18.0,23.7) |  | 17.3(15.4,19.4) | 17.9(14.6,21.8) |
| 4st quintile | 21.9(19.4,24.7) |  | 21.4(19.2,23.7) | 21.1(17.5,25.3) |
| 5st quintile | 24.7(21.5,28.1) |  | 28.1(25.6,30.9) | 25.8(21.5,30.7) |
| **Mother living with a partner** |  |  |  |  |
| Não | 9.7(7.9,11.7) |  | 15.4(13.7,17.4) | 14.4(11.7,17.8) |
| Sim | 90.3(88.3,92.0) |  | 84.6(82.7,86.3) | 85.5(82.2,88.3) |
| **Area of residence** |  |  |  |  |
| Urbana | 59.2(55.0,63.3) |  | 58.4(55.8,60.9) | 65.6(60.7,70.1) |
| Rural | 40.8(36.7,44.9) |  | 41.6(39.0,44.2) | 34.4(29.9,39.3) |
| **Mother working outside of home** |  |  |  |  |
| Não | 65.6(62.5,68.7) |  | 60.7(58.1,63.2) | 66.2(61.8,70.4) |
| Sim | 34.4(31.3,37.5) |  | 39.3(36.8,41.9) | 33.8(29.6,38.2) |
| **Breastfed in the first hour** |  |  |  |  |
| Não | 55.8(52.5,59.1) |  | 47.9(45.4,50.5) | 44.3(39.8,48.9) |
| Sim | 44.2(40.9,47.5) |  | 52.0(49.5,54.6) | 55.7(51.1,60.2) |
| **C-section** |  |  |  |  |
| Não | 90.1(87.7,92.0) |  | 83.7(81.4,85.8) | 72.3(67.9,76.3) |
| Sim | 9.9(7.9,12.3) |  | 16.3(14.2,18.6) | 27.7(23.7,32.1) |
